# Friends With Benefits: Exploring the Phycosphere of the Marine Diatom *Skeletonema marinoi*

**DOI:** 10.3389/fmicb.2019.01828

**Published:** 2019-08-06

**Authors:** Oskar N. Johansson, Matthew I. M. Pinder, Fredrik Ohlsson, Jenny Egardt, Mats Töpel, Adrian K. Clarke

**Affiliations:** ^1^Department of Biological and Environmental Sciences, University of Gothenburg, Gothenburg, Sweden; ^2^Department of Marine Sciences, University of Gothenburg, Gothenburg, Sweden; ^3^Department of Mathematical Sciences, Chalmers University of Technology, University of Gothenburg, Gothenburg, Sweden; ^4^Gothenburg Global Biodiversity Centre, Gothenburg, Sweden

**Keywords:** bacteria, diatoms, microbiome, marine, symbiosis

## Abstract

Marine diatoms are the dominant phytoplankton in the temperate oceans and coastal regions, contributing to global photosynthesis, biogeochemical cycling of key nutrients and minerals and aquatic food chains. Integral to the success of marine diatoms is a diverse array of bacterial species that closely interact within the diffusive boundary layer, or phycosphere, surrounding the diatom partner. Recently, we isolated seven distinct bacterial species from cultures of *Skeletonema marinoi*, a chain-forming, centric diatom that dominates the coastal regions of the temperate oceans. Genomes of all seven bacteria were sequenced revealing many unusual characteristics such as the existence of numerous plasmids of widely varying sizes. Here we have investigated the characteristics of the bacterial interactions with *S. marinoi*, demonstrating that several strains (*Arenibacter algicola* strain SMS7, *Marinobacter salarius* strain SMR5, *Sphingorhabdus flavimaris* strain SMR4y, *Sulfitobacter pseudonitzschiae* strain SMR1, *Yoonia vestfoldensis* strain SMR4r and *Roseovarius mucosus* strain SMR3) stimulate growth of the diatom partner. Testing of many different environmental factors including low iron concentration, high and low temperatures, and chemical signals showed variable effects on this growth enhancement by each bacterial species, with the most significant being light quality in which green and blue but not red light enhanced the stimulatory effect on *S. marinoi* growth by all bacteria. Several of the bacteria also inhibited growth of one or more of the other bacterial strains to different extents when mixed together. This study highlights the complex interactions between diatoms and their associated bacteria within the phycosphere, and that further studies are needed to resolve the underlying mechanisms for these relationships and how they might influence the global success of marine diatoms.

## Introduction

Silica-encrusted diatoms are ubiquitous marine primary producers that globally dominate the temperate oceans and coastlines. Together with other phytoplankton, they contribute around half of the total CO_2_ fixation on earth (Field et al., 1998). The diatom hallmark, its silicated shell or frustule, is covered with secreted exopolymeric substances (EPS) causing them to aggregate and sink to the ocean floor as “marine snow,” which is an integral part of the ocean cycling of carbon and other nutrients (Fowler and Knauer, 1986; Passow and Alldredge, 1995; Simon et al., 2002). In close proximity to the cell surface of diatoms is a diffusion gradient, the phycosphere, where key nutrients are exchanged between the diatom and the surrounding bacteria (Bell and Mitchell, 1972; Amin et al., 2012). Formation of this micro-environment beneficial for microbial interaction is promoted by EPS. It is known that co-culturing of diatoms with specific marine bacteria accelerates the flocculation process by increasing EPS production and changing its composition (Grossart, 1999; Sonnenschein et al., 2011). This is especially important since aggregation is a key event at the end of di-annual diatom blooms (Alldredge and Gotschalk, 1989; Liu et al., 2008; Park et al., 2010, 2015).

Ocean-living bacteria, like other life forms, are in constant need of organic carbon and are either passively or actively pursuing sequestration of such compounds from photosynthetic primary producers such as diatoms. The interaction between these organisms ranges from the purely symbiotic to parasitic and opportunistic, including passive acquaintances that only feed upon dead or dying diatom cells (Grossart, 1999; Grossart et al., 2005). This flux of organic carbon from phytoplankton to marine bacteria forms the microbial loop through which no less than half of all fixed carbon flows through the bacteria (Azam et al., 1983). Microbes that proliferate in close proximity to the diatom differ significantly from those that live outside of the diatom-EPS biofilm (Delong et al., 1993). Those interacting bacteria are well adapted to a narrow range of compounds secreted by their diatom host, forming an exclusiveness that contributes to their specific association (Bell, 1984; Schafer et al., 2002; Sison-Mangus et al., 2014). Within the diatom phycosphere, there are examples of symbiotic bacteria providing the host with vitamins and organic acids, including amino acids and siderophores (Amin et al., 2009). Diatoms in return provide the bacteria with easily accessible carbon- and sulfur sources (Seymour et al., 2017).

The diatom lineage has a complex evolutionary history with extensive horizontal gene transfer between diatom and bacteria, suggestive of a long-term association within the phycosphere (Armbrust et al., 2004; Bowler et al., 2008; Mock et al., 2017). Despite this, the contribution of each interacting bacterial species within the phycosphere and the level of functional overlap between them remains unclear. Equally uncertain is the underlying genetic basis for the host-microbiome interaction and if this mechanism is conserved between pennate and centric diatoms. In this study, we have investigated the different bacteria that constitute the microbiome of the centric marine diatom *Skeletonema marinoi*. *Skeletonema* is a prolific genus in the temperate oceans and coastal waters where diatoms dominate the phytoplankton communities. In Scandinavian waters, *S. marinoi* is a crucial primary producer that occurs throughout the year, especially during spring when it forms extensive blooms (de Vargas et al., 2015). *S. marinoi* also represents the obligate chain-forming diatoms that play a central role in the oceanic biogeochemical cycles that sequester and mineralize carbon, nitrogen and silica, as well as forming resting stages that are revivable even after a century (Härnstrom et al., 2011). We have recently proposed *S. marinoi* as a new genetic model for chain-forming diatoms, having sequenced its genome and established a collection of randomly mutagenized but identifiably tagged strains suitable for large-scale phenotyping (Johansson et al., 2019). We have now also isolated several different bacteria from *S. marinoi* that has been in continuous culture for nearly a decade and determined the effect each individual bacterial species has on their diatom host.

## Materials and Methods

### Cell Isolation and Culture Conditions

*Skeletonema marinoi* strains ST54 and R05AC were maintained in f/2 + Si medium under standard growth conditions (16°C, 16 h photoperiod with an irradiance of 75 μmol photons m^–2^ s^–1^ from LED-light panels (Heliospectra, Sweden). Both strains are also available from the Gothenburg University algal culture bank^1^. To isolate any bacterial species growing in association with *S. marinoi* in culture, aliquots were plated onto marine agar 2216 (BD, United States) and incubated at 16°C in darkness until colonies appeared. This procedure was performed on multiple occasions and the plates incubated over different time periods in order to maximize the range of bacterial species isolated. Colonies were then subjected to multiple iterations of serial dilution streaking until single bacterial isolates could be distinguished, after which they were maintained on marine agar plates with regular re-streaking.

### DNA Extraction and Sequencing

Cultures of each bacterial strain (50 mL) were grown overnight at 30°C in marine broth 2216 (BD, United States). Cells were later pelleted and ground in liquid nitrogen, with genomic DNA then extracted using Plant DNAzol (Thermo Fisher Scientific, United States) according to the manufacturer’s instructions. A region of the highly conserved 16S rRNA gene was PCR amplified using a degenerate forward primer (16S_fwd, 5′-ATTAGITWGTTGGTRRGGTAA-3′) and one of the two different specific reverse primers (16S_rev1, 5′-CGGCTGCTGGCACGGAGTTAG-3′, 16S_rev2, 5′-CCACATGCTCCACCGCTTGTG-3′) along with the AmpliTaq DNA polymerase (Thermo Fisher Scientific, United States). Amplicons were purified using a Wizard PCR purification kit (Promega, United States) and Sanger sequenced (Eurofins Genomics, Germany).

### *Skeletonema marinoi* Growth Assays

*Skeletonema marinoi* isolate R05AC was pre-cultured in replicate cultures (*n* = 8) for five *d* in enriched growth media, EGM (Johansson et al., 2019). Cultures were centrifuged (1500 × *g*, 5 min), washed once in fresh EGM, and dissolved in 20 mL EGM. Cell density of washed pre-cultures was measured (Gross et al., 2018) and then used to prepare experimental cultures with a starting concentration of 25 000 cells mL^–1^. Bacterial cultures (5 mL) were grown in Difco marine broth (BD Biosciences, United States) overnight with shaking a day before experiment. Bacterial cells were pelleted (4000 × *g*, 7 min), washed in 10 mL EGM and again pelleted, then dissolved in 3 mL of EGM. OD_600_ was measured on a diluted aliquot and the cell concentration was based on OD_600_ = 1 that equates to 5 × 10^8^ cells mL^–1^. Growth assays were performed under standard conditions in Falcon 48-well tissue culture plates (Corning, United States) unless otherwise stated and chlorophyll fluorescence was monitored daily. Average growth was measured as relative fluorescence units and normalized at time zero, with the growth rates from time 0 to 24 h (designated α) and 24 to 48 h (β) determined from the slope of the growth curve. Low iron condition was set as 15% of EGM (49 to 7.2 μM). Light quality shifts were performed by covering the growth plates with plexiglass of the indicated color, which reduced the light intensity to 25–30 μmol photons m^–2^ s^–1^. Growth assays using various chemical additions were performed as above, except for four replicate pre-cultures being used instead of eight. The chemicals, their respective vendors, stock solvent and concentration are shown in Supplementary Table S1. Antibiotic sensitivity tests were performed by supplementing *S. marinoi* cultures (*n* = 6) with the respective antibiotics at the indicated concentrations prepared from stocks (Supplementary Table S1), with growth measured by chlorophyll fluorescence for four *d*.

### Bacterial Growth Assays and Characterization

Antibiotic resistance and *in vitro* growth inhibition assays were performed using the disc diffusion method (Davis and Stout, 1971). In short, filter paper discs (Ø = 5 mm) were sterilized by autoclaving, submerged in either the antibiotic or respective bacterial suspension, and then placed on a marine agar plate that had previously been covered with a thin layer of bacterial suspension (OD_600_ = 0.05). Plates were incubated at 30°C overnight, with growth inhibition then scored the following day as none, minor, moderate or severe. Assessment of growth capability in terms of carbon sources was performed using BIOLOG III (Bochner, 1989) plates in accordance with the manufacturer’s instructions. Most strains were assayed on the BIOLOG III plates using the standard IG-A solution, but due to the reductive nature of a subset of bacterial isolates IG-B had to be used.

### Chlorophyll Fluorescence

Co-cultures (*n* = 8) of the respective bacteria and *S. marinoi* R05AC were prepared in Falcon 48-well tissue culture plates from pre-cultures using the same procedure as for the growth assays. After growth for three *d* under standard conditions, the plate was placed into an Imaging PAM (PSI, Czechia), dark-adapted for 1 h and photosynthetic performance as F_*V*_/F_*M*_ was determined.

### Bioinformatics Analysis

The genomes of each bacterial strain were assembled using either of the assembly programs Falcon version 1.7.5^2^ (Gordon et al., 2016) or Canu version 1.3 (Koren et al., 2017), and annotated using Prokka version 1.12 beta (Seemann, 2014). A phylotaxonomic analysis was performed for each strain independently using PhyloPhlAn version 0.99 (Segata et al., 2013). The taxonomic classification was also supported by marker gene comparisons, as well as by analysis of physical characteristics of the bacterial cells and colonies. The presence of prophages in each genome was determined using the phage search tool PHASTER^3^ (Arndt et al., 2016). The presence of genes involved in biotic-related interactions was ascertained using both Pathway Tools version 20.5/21.0 (Karp et al., 2002), and antiSMASH version 3.0.5^4^ with all optional analyses turned on (Weber et al., 2015), as well as manual searches of each strain’s annotation. The process is described in more detail in each strain’s respective genome announcement article (Töpel et al., 2017, 2018a,b, 2019a,b; and two additional papers in preparation).

### Statistical Analysis

Statistical analysis was performed in the software Prism 8 (GraphPad Software Inc., United States).

## Results

### Bacterial Strains Cultured From *S. marinoi*

Non-axenic isolates of the diatom *S. marinoi* have been in continuous culture in the Gothenburg University algal bank since their isolation in 2010. During the early stages of sequencing of the *S. marinoi* genome, DNA fragments from multiple bacterial species were present within the genomic data (Töpel et al. in preparation). This, along with microscope observations led us to believe that several bacterial strains coexisted in the diatom cultures, even after almost a decade of laboratory culturing. To determine the composition of this microbiome, two *S. marinoi* isolates (R05AC and ST54) were streaked onto separate marine agar plates and incubated in darkness. Iterative dilution streaking produced identifiable single-strain bacterial isolates, with the genus for each determined by sequencing partial 16S regions. Seven different genera were eventually isolated with multiple species present, one of which, from each genus, was selected as a representative for further study (Supplementary Figure S1) including whole genome sequencing (Töpel et al., 2017, 2018a,b, 2019a,b).

### Bacterial Stimulation of Diatom Growth

**FIGURE 1:**
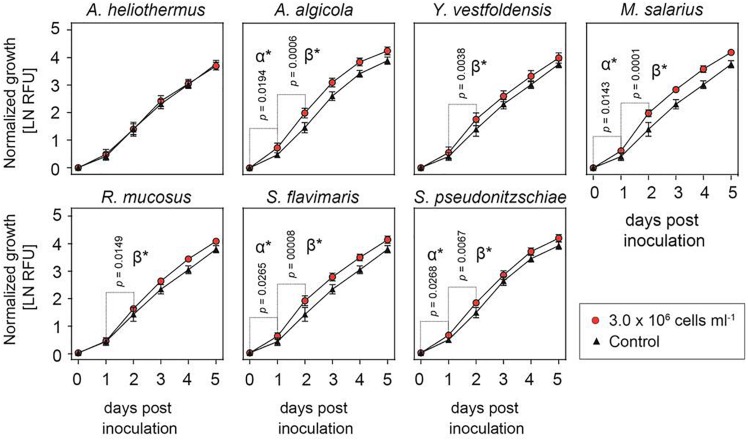
Growth-enhancing capacity of isolated bacterial cultures. Growth of *Skeletonema marinoi* cultures with (red circles) and without (black triangles) addition of the indicated bacterial species. Shown is average growth normalized at time zero and measured as relative fluorescence units, RFU (*n* = 8, ±SD).

Despite numerous attempts to establish axenic cultures of the R05AC isolate of *S. marinoi*, all failed in part due to this diatom’s exceptional sensitivity to a wide variety of antibiotics and the inability of the antibiotic cocktails to remove all bacteria from the cultures (Supplementary Figures S2, S3). Given the likelihood that most if not all of the bacterial strains isolated from the *S. marinoi* cultures are in some form of symbiotic relationship, some of which could be important for the viability of the diatom host, we tested if adding one bacterial species in excess to the non-axenic culture affected growth of *S. marinoi* under standard conditions (Figure 1). Equivalent abrupt shifts in microbiome composition would almost certainly occur in nature during diatom blooms or other sudden stochastic environmental changes. As shown in Figure 1, the growth rate of *S. marinoi* was significantly enhanced throughout the first 48 h after the addition of *Arenibacter algicola*, *Marinobacter salarius*, *Sphingorhabdus flavimaris* or *Sulfitobacter pseudonitzschiae* (designated rate α, 0–24 h, and rate β, 24–48 h), whereas such growth stimulation occurred only in the second half (rate β) after addition of *Yoonia vestfoldensis* or *Roseovarius mucosus*.

**FIGURE 2:**
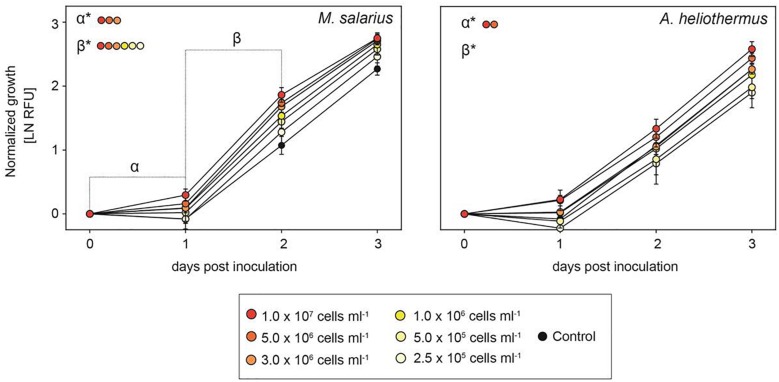
The growth-promoting effect of added bacteria to *Skeletonema marinoi* cultures. Relative growth of *S. marinoi* after the addition of either *M. salarius* or *A. heliothermus* at different cell densities, with growth rates normalized at time zero and measured as RFU (*n* = 4, ±SD). Significance between slopes was calculated using multiple *t*-tests. *P*-values were adjusted for multiple testing using the Sidak–Bonferroni method, with asterisks indicating significant changes (adjusted *p* < 0.05).

**FIGURE 3:**
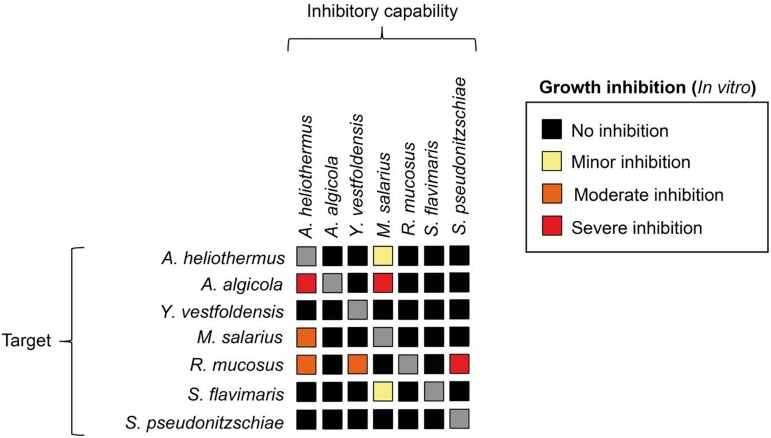
The capacity of bacterial species to inhibit each other’s growth. Each bacterial specie was tested using the disc diffusion method. Bacterial cell-suspensions were dispersed to cover the entirety of a marine agar plate. Filter discs were submerged in a cell-suspension of each respective bacterial species, wiped and then one disc from each bacterial strain was put on each agar plate. The inhibitory capacity was scored as minor (yellow), moderate (orange) or severe (red) based on the zone of inhibition around the filter disc.

The growth-promoting effect of the added bacteria was positively correlated to the bacteria/diatom cell ratio, as evidenced by the greater growth-promoting *M. salarius* and lesser growth-promoting *Antarctobacter heliothermus* (Figure 2). A threshold was specifically reached for *M. salarius/S. marinoi* when adding more than 3 × 10^6^ bacterial cells per mL, corresponding to a bacteria-to-diatom ratio of 100:1 (Amin et al., 2015). At this ratio and higher, growth stimulation could be seen already after the first 24 h. A similar effect was evident also for *A. heliothermus*, which promoted growth within the first 24 h at a threshold concentration of 5 × 10^6^ bacterial cells per mL. It should be noted that no additional stimulation of *S. marinoi* growth occurred after 48 h with any of the added bacteria. Also potentially complicating the growth enhancement effects was the capability of certain bacteria to inhibit the growth of other bacteria from the *S. marinoi* microbiome, pointing to a more intricate relationship between the isolated bacteria within the phycosphere (Figure 3). These complex interactions presumably re-establish inter-bacterial and diatom–bacterial homeostasis and resumption of growth comparable to that of untreated *S. marinoi* post 48 h of co-culture.

### Environmental Conditions Shape the Diatom–Bacterial Interactions

**FIGURE 4:**
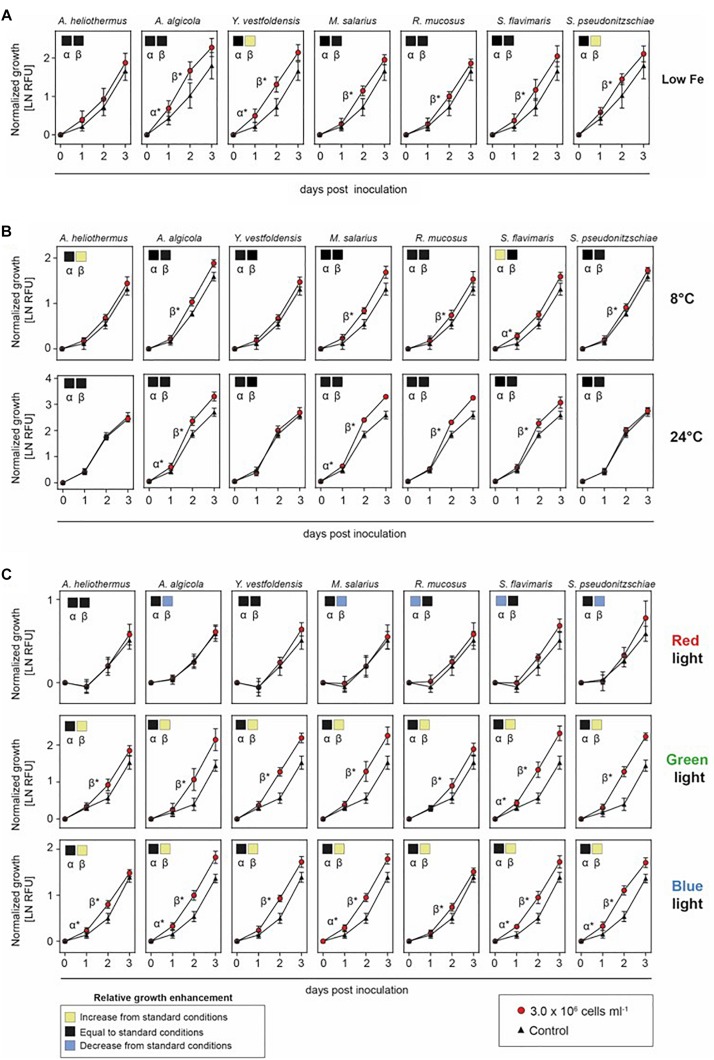
Growth enhancement under variable iron, temperature and light conditions. Growth of *Skeletonema marinoi* cultures in co-culture with indicated bacterial species. Shown are growth rates normalized at time zero and measured as RFU (*n* = 8, ±SD) of the control (triangles) and co-cultures (circles) under different ecologically relevant low iron concentration **(A)**, temperatures **(B)**, and light qualities **(C)**. Significance between slopes was calculated using multiple *t*-tests. *p*-values were adjusted for multiple testing using the Sidak–Bonferroni method. Significant increases in *S. marinoi* growth by the addition of a specific bacterial species (adjusted *p* < 0.05) are indicated by either α^*^ (0–24 h) or β^*^ (24–48 h). Square boxes display a comparison of growth rates during the α- and β-phases to the standard nutrient-replete growth conditions, with yellow indicating a significant enhancement, black unchanged and blue a significant decrease as determined by one-way ANOVA with corrections for multiple comparisons using the Bonferroni method.

The interaction between diatoms and their associated bacteria is influenced by the physical constraints of the marine environment, including temperature and in particular nutrient availability (Gardes et al., 2012; Meyer et al., 2016). The major nutrients needed for diatom growth consist of nitrogen, phosphorus, silica and iron. Filtered and autoclaved deep ocean water with nutrient and mineral enrichments has been the media of choice when growing many marine phytoplankton (Guillard, 1975). While *S. marinoi* grew well for limited periods in artificial seawater, it eventually proved unreliable as cultures would randomly die intermittently. Although the standard f/2 + Si growth media commonly used for most marine diatoms as well as artificial sea water did support the enhanced growth of *S. marinoi* after the addition of *M. salarius* but not *A. heliothermus* (Supplementary Figure S4), the further enriched media EGM as previously described (Johansson et al., 2019) always resulted in a more reliable effect, consistent with earlier findings that low nutrient conditions can attenuate symbiotic benefits (Gardes et al., 2012; Meyer et al., 2016). In light of this, we examined if one or more of the bacteria had a beneficial effect on *S. marinoi* growth under low iron concentration (15% of that in standard f/2 + Si media), an often limiting factor in oceans for most phytoplankton (Martin and Gordon, 1988; Rue and Bruland, 1995). Interestingly, it was only *Y. vestfoldensis* and *S. pseudonitzschiae* that had some additional benefit under low iron concentrations during the β growth rate of *S. marinoi* (Figure 4A).

We next examined if other environmental variables could influence the diatom–bacterial interactions. Shifting temperatures are associated with ocean depth and the changing seasons throughout the year. As shown in Figure 4B, the effect of growth temperature on the interaction was strongest at the low temperature (8°C), where the *S. marinoi* interactions with *A. heliothermus* and *S. flavimaris* were especially favorable. In contrast, high temperature (24°C) had little effect on the interaction with the different bacteria compared to the standard growth temperature (16°C). Another environmental factor that varies with ocean depth is light quality, with blue light penetrating far deeper than the increasingly longer visible wavelengths of green and red. By illuminating the diatom under co-culture with specific color ranges of the visible light spectrum, we next tested the effect of light quality on the interaction as would be expected at different depths within the ocean water column. As shown in Figure 4C, *S. marinoi* grew poorly under red light and all bacteria when added had little or no stimulatory effect upon the diatom growth rate. In contrast, *S. marinoi* grew far better under either green or blue light than under red light and the addition of each bacterial species enhanced the growth rates, especially during the β-phase.

### Mechanics of the Diatom–Bacteria Interaction Do Not Resemble Those of Plant Symbioses

Since a comprehensive model of the interaction of diatoms with their associated bacteria is currently lacking, we compared this interaction to those well-characterized ones between plants and different microbial partners. For example, symbiotic interactions between land plants and mycorrhiza-forming fungi are thought to enhance photosynthetic performance (Schweiger et al., 2014), but no such improvement in photosynthetic performance of *S. marinoi* was observed after the addition of each of the bacteria acting within the phycosphere under standard growth conditions (data not shown). Certain important similarities are known to exist between the diatom/bacteria interaction in the phycosphere and that between plants and diazotrophic bacteria of the rhizobium, such as the ability of the bacteria to provide the host with iron (Carson et al., 1992). Another is the use of plant hormones (Grunewald et al., 2009), with evidence that auxins including indole-3-acetic acid (IAA) and a structurally related compound, indole-3-acetonitrile (IAN), are produced by bacteria within the phycosphere to stimulate growth of their diatom partner (Amin et al., 2015). Analysis of the *S. marinoi* reference genome does reveal genes encoding most of the enzymes functioning within the auxin biosynthetic pathway as defined in plants. Despite this, only indole (IND), one of the early auxin precursors, had a growth stimulatory effect on *S. marinoi* cultures, with no such effect produced by any of the nine naturally occurring or synthetic auxins, nor by the addition of the amino acids tyrosine (TYR) and cysteine (CYS) or the phytoplankton-secreted molecule taurine (TAU) (Supplementary Figure S5A). Moreover, the addition of tryptophan (TRP), IAA or IAN had no effect on the interactions between *S. marinoi* and either the growth-promoting *M. salarius* or non-growth-promoting *A. heliothermus* (Supplementary Figure S5B).

**FIGURE 5:**
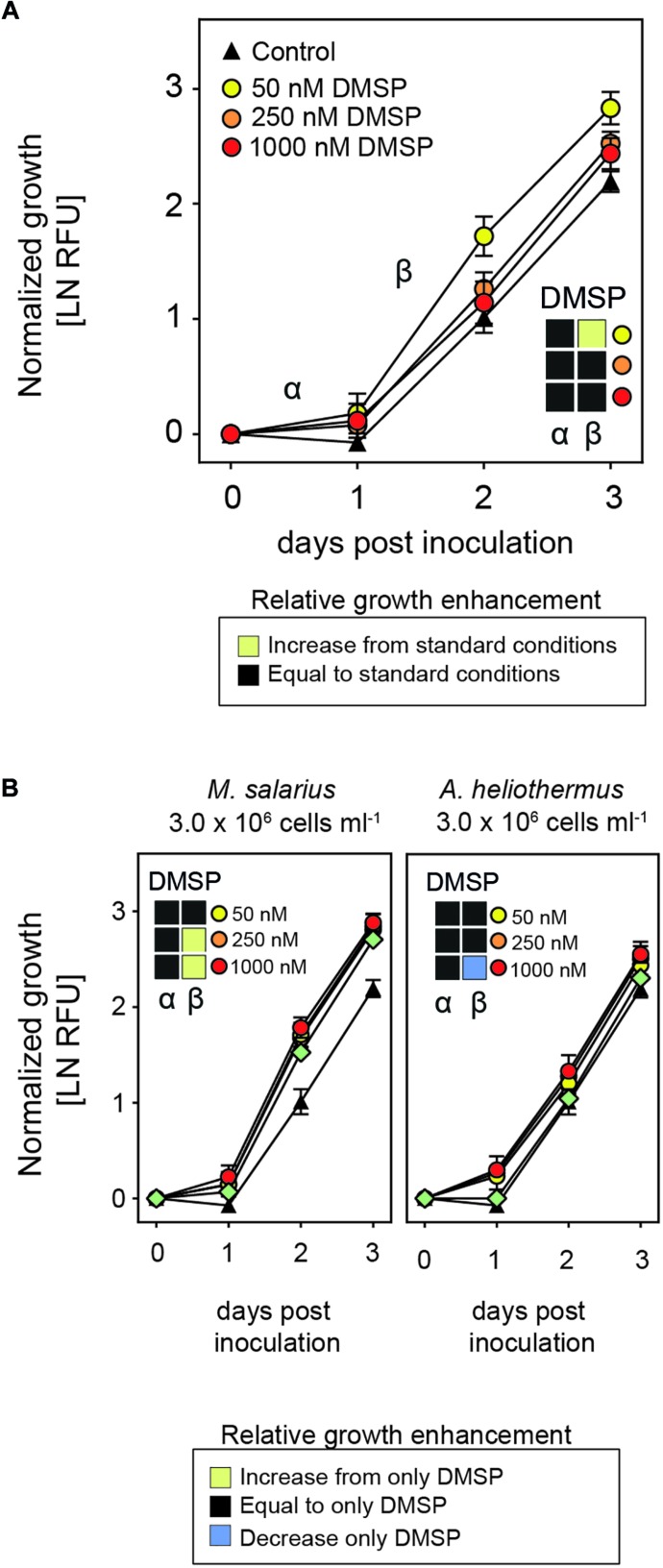
Growth-enhancing capacities of DMSP. **(A)** Growth of *Skeletonema marinoi* cultures after the addition of different concentrations of DMSP (50 nM – yellow circles, 250 nM – orange circles and 1000 nM – red circles), with the control being *S. marinoi* with no added DMSP (black triangles). Shown are growth rates during the α- and β-phases normalized at time zero and measured as RFU (*n* = 4, ±SD). The box inserts display a comparison of growth rates relative to the standard growth conditions (control) during the respective time points as indicated by color. Significant changes (adjusted *p* < 0.05) in slopes were determined by multiple *t*-tests with Sidak–Bonferroni correction for multiple comparisons. **(B)** Change in growth rate of *S. marinoi* cultures after addition of both DMSP at indicated concentrations (50 nM – yellow, 250 nM – orange and 1000 nM – red circles) and either *M. salarius* or *A. heliothermus*, plus bacterial addition alone (green diamonds) compared to that of DMSP only. Growth rates during the α- and β-phases were normalized at time zero and measured as RFU (*n* = 4, ±SD). The box inserts display a comparison of growth rates relative to the standard growth conditions (control) during the respective time points as indicated by color. Significant changes (adjusted *p* < 0.05) in slopes were determined by multiple *t*-tests with Sidak–Bonferroni correction for multiple comparisons. Yellow shading indicates a significantly higher growth enhancement, blue a significantly reduced growth enhancement, and black no significant change.

Since the interaction between *S. marinoi* and most of its bacterial partners is synergistic, we next examined the potential role of dimethylsulfoniopropionate (DMSP), a compound known to be produced by diatoms both as an intercellular osmolyte and as a compound secreted into the phycosphere and used by the surrounding bacteria (Moran et al., 2012; Buchan et al., 2014; Amin et al., 2015). Growth enhancement of the diatom provoked by the addition of DMSP would be mediated by the various bacteria within the phycosphere, suggesting that the diatom–bacteria interaction in standard non-axenic cultures is limited. To test if this was the case, DMSP was added to cultures of *S. marinoi* at different concentrations in a range that was ecologically relevant (Aumont et al., 2002). Interestingly, there was a stimulatory effect on *S. marinoi* growth at the lowest concentration of DMSP used (i.e., 50 nM) but not at higher concentrations (Figure 5A). We next combined the addition of DMSP with that of the growth-promoting *M. salarius* and observed significant growth enhancement of *S. marinoi* at the higher concentrations of DMSP (i.e., ≥250 nM), whereas substituting *M. salarius* with the non-growth-promoting *A. heliothermus* attenuated the DMSP growth-enhancing effect (Figure 5B).

## Discussion

Laboratory cultures of diatoms tend to keep many of their associated bacteria, especially those in some form of symbiotic relationship if not treated with antibiotics. The relationship for many diatoms has proven so intertwined that even exhaustive attempts to remove them sometimes fail as was the case with *S. marinoi* in this study. We were able to eventually isolate seven strains of bacteria from stock cultures of *S. marinoi* that had been kept in culture for almost a decade. By shifting the bacterial equilibrium within the *S. marinoi* cultures we were able to study the effect of each individual bacterial species on the diatom host. Additions of each bacterium to the cultures revealed that all but one (*A. heliothermus*) strongly stimulated the growth of *S. marinoi*. The growth enhancement effect was dependent on time and the diatom to bacteria ratio, with the two bacterial species that most consistently promoted growth under the different conditions tested being *M. salarius* and *A. algicola*.

*Marinobacter* sp. are commonly found associated with algal field samples and remain even in decade-old cultures of *Skeletonema costatum* (Jian et al., 2019). The growth-affecting properties (both positive and negative) of the *Marinobacter* genus have been previously demonstrated by co-culturing with diatoms such as *S. costatum* and *Thalassiosira pseudonana* (Grossart et al., 2005, 2006; Grossart and Simon, 2007; Yang et al., 2013). Interestingly, *Marinobacter* sp. either enhanced growth as in the case of *T. pseudonana* or reduced it as in *S. costatum* compared to axenic cultures during the first days of co-culture. In contrast, the *M. salarius* strain identified in our cultures significantly promoted the growth of *S. marinoi*, attenuated only by red light, suggesting that even at the species level there is exclusiveness within the phycosphere environment for each diatom species. Similarly, members of the *Arenibacter* genus are also known to stimulate growth of the diatoms *T. pseudonana* and *Phaeodactylum tricornutum* (Zecher et al., 2015). This was indeed also true for *A. algicola* in this study, promoting the growth of *S. marinoi* with similar characteristics to that by *M. salarius*, although again with the exception of red-light conditions. It should be noted that the relative lack of growth stimulation of *S. marinoi* by these bacteria under red light is likely linked to limited light absorption of these visible light wavelengths and the resulting minimal photosynthetic activity and overall growth.

The low iron concentration experiment revealed specific benefits of certain bacteria. Soluble iron (III) in oceans is relatively scarce and is often the limiting factor for growth of most phytoplankton (Martin and Gordon, 1988; Rue and Bruland, 1995). Iron supplementation is also important in certain terrestrial habitats such as for the symbiotic interaction between plants and bacteria within the rhizobium (Berraho et al., 1997). In the marine environment, certain bacteria can also produce iron-chelating agents, including siderophores, which aid in iron assimilation. In general, the absence of such chelating agents could pose potential problems for the intrinsic relationship between diatoms and bacteria in a low iron environment. Interestingly, genes encoding siderophores are present in the genomes of the *M. salarius* and *S. pseudonitzschiae* (Töpel et al., 2019a) strains from our study (Supplementary Figure S5). Under low iron conditions, addition of *S. pseudonitzschiae* did indeed enhance *S. marinoi* growth under otherwise standard growth conditions whereas there was no additional effect on the already strong growth-promoting *M. salarius*, the lack of which suggests another constraint being limiting. In contrast, co-culture of *S. marinoi* with *Y. vestfoldensis* also improved growth under low iron conditions compared to standard conditions, despite the lack of recognizable genes encoding siderophores within the *Y. vestfoldensis* genome, opening the possibility for other chelating agents being produced.

Ocean water temperatures and light conditions shift throughout the year and with water depth. Interestingly, low temperature (8°C) enhanced the growth stimulation on *S. marinoi* cultures but only with the addition of *A. heliothermus* and *S. flavimaris*, whereas no significant change was observed at high temperatures (24°C) relative to the standard temperature (16°C). At this stage, the reason for this beneficial effect of *A. heliothermus* and *S. flavimaris* at low temperatures on *S. marinoi* growth remains unclear, with no obvious clues from the genome comparisons between the different bacteria. In comparison, changes in light quality did significantly alter the stimulatory response by each bacterial strain on *S. marinoi* growth. The fact that green and blue, but not red light produced the stimulatory response suggests it can be limited by reduced photosynthetic activity of the diatom host. Given that the pigment composition of diatoms consists of chlorophylls and various carotenoids that together absorb higher proportions of blue/green light, the improved photosynthetic rates and thereby growth under these light conditions would place fewer limitations on the stimulatory effect the added bacteria have on *S. marinoi* cultures. Conversely, lower light absorption and subsequent reduced photosynthetic activity under red light would likely be rate-limiting on overall growth of *S. marinoi*, which would therefore mask any potential benefit from the addition of the bacterial strains.

One group of signaling molecule that is known to influence the growth of certain diatoms and other eukaryotic phytoplankton is the auxin IAA and its derivatives (Amin et al., 2015; Labeeuw et al., 2016). Auxins are best known as an important group of plant hormones responsible for a wide range of growth and developmental changes such as cell expansion, tissue differentiation and fertility (Zhao, 2010). Besides plants, certain bacteria and fungi also synthesize auxins (Spaepen et al., 2007) although much of their function in these organisms has been inferred only from the extensive studies done in plants. More recently, synthesis of IAA was shown in a *Sulfitobacter* sp. in response to an increase in tryptophan synthesis by its diatom host, the pennate *Pseudo-nitzschia multiseries*, which in turn stimulated the growth of the diatom (Amin et al., 2015). Interestingly, the lack of any growth stimulatory effect by IAA or other auxin-related compounds in *S. marinoi* cultures as shown in this study suggest a possible functional split between centric- and pennate diatoms regarding the chemical interactions within their respective phycospheres. Indeed, the absence of a recognizable plant-like auxin response mechanism mediated by ARF transcription factors in the genome of sequenced diatoms further suggests that much remains unknown about the role of auxins and other signaling compounds within the phycosphere of different diatoms.

In contrast to auxins, DMSP did promote growth of *S. marinoi* cultures within a certain concentration range, with the concentration dependence being explained by the addition of either *M. salarius* or *A. heliothermus* together with DMSP. DMSP is typically excreted by diatoms and taken up by associated bacteria, after which it is degraded to different organosulfur compounds (Moran et al., 2012). Interestingly, the genes required for DMSP degradation are present in the genomes of all but two of the seven bacteria isolated from *S. marinoi* cultures (Supplementary Figure S6). In *S. marinoi* cultures with the normal proportion of associated bacterial species, the lower concentrations of DMSP were likely suitable for stimulating growth of the bacteria, which in turn further promoted growth of *S. marinoi*, with higher concentrations of DMSP possibly in excess and outside of the optimal range. In contrast, the stimulatory effect of DMSP was only observed at the higher concentrations after the addition of *M. salarius*, most likely due to the greater proportion of bacterial cells within the culture and the need for extra DMSP to promote their growth. Besides DMSP, additional compounds are also likely secreted by diatoms in order to stimulate growth of bacteria in the phycosphere. A range of carbohydrates and amino acids have been suggested to be involved (Bruckner et al., 2008, 2011) and most bacteria isolated from *S. marinoi* cultures could utilize one or more forms of carbohydrates, although there did appear a preference for amino acids and other organic acids (Supplementary Figure S7).

It is well known that genomes of uncultivatable bacteria often occur in metagenomic data (Vartoukian et al., 2010), suggesting that the phycosphere of *S. marinoi* likely includes more bacterial species than those isolated in this study. Nevertheless, our experiments provide ample evidence that growth-enhancing properties remain among the associated bacteria isolated from laboratory cultures even after a decade of being isolated from nature, and that these differ significantly from those between plants and their symbionts. Indeed, growth addition experiments have been performed with similar results on numerous occasions over the course of several years, comprising hundreds of bacterial generations, suggesting there remains a strong selective pressure for maintaining these different bacterial species within the diatom phycosphere.

Although most of the bacterial species isolated from *S. marinoi* cultures had a positive effect on diatom growth, understanding the presence of *A. heliothermus* was more elusive. It would on occasion, such as with low temperature and different light quality, improve *S. marinoi* growth to some extent but overall it had little significant effect. It should be noted, however, that this species was only found in the *S. marinoi* isolate ST54 and not in the R05AC isolate used for much of this study. Although the two isolates are very similar in terms of genome sequence, this difference in bacterial composition could indicate that the phycosphere in each *S. marinoi* isolate are more specialized and fine-tuned than previously thought.

## Data Availability

The datasets generated for this study are available on request to the corresponding author.

## Author Contributions

OJ performed most of the experimental work, analyzed the experimental data, and wrote the manuscript. MT and MP provided bioinformatic expertise and sequence analyses. JE helped with the growth assays. FO assisted in growth rate analyses. AC designed the project, analyzed the experimental data, and wrote the manuscript. All authors helped in finalizing the manuscript.

## Conflict of Interest Statement

The authors declare that the research was conducted in the absence of any commercial or financial relationships that could be construed as a potential conflict of interest.

## Footnotes

1 https://marine.gu.se/english/research/marine-biology/algal-bank

2 https://github.com/PacificBiosciences/FALCON/

3 http://phaster.ca

4 https://antismash.secondarymetabolites.org/
