# Supplementary material for: Friends With Benefits: Exploring the Phycosphere of the Marine Diatom Skeletonema marinoi
Source: Front Microbiol. 2019 Aug 6;10:1828. doi: 10.3389/fmicb.2019.01828 (PMC6691348; doi:10.3389/fmicb.2019.01828)
Supplement: Supplementary file 1 [file Data_Sheet_1.PDF]

Friends with Benefits: Exploring the Phycosphere of the Marine Diatom *Skeletonema marinoi*

Oskar N. Johansson[1], Matthew I.M. Pinder[2], Fredrik Ohlsson[3,†], Jenny Egardt[1], Mats Töpel[2,4] and Adrian K. Clarke[1*]

SUPPLEMENTARY FIGURES

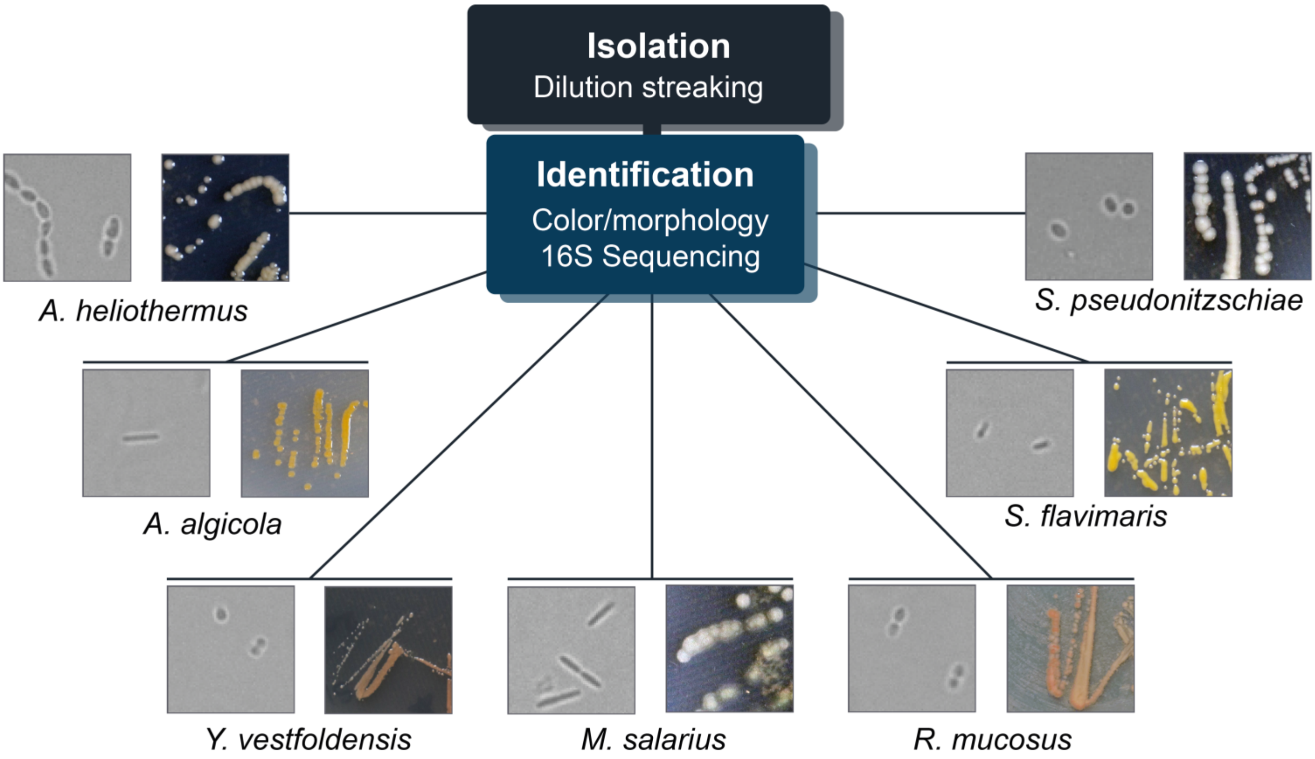

Figure S1. Isolated bacterial species. Bacteria were isolated using dilution streaking and identified based on coloration, morphology and 16S rRNA gene sequencing. Also shown are the colorations of the isolated bacteria when grown on marine agar plates and to the left of that their appearance under light microscope at 1000 X magnification.

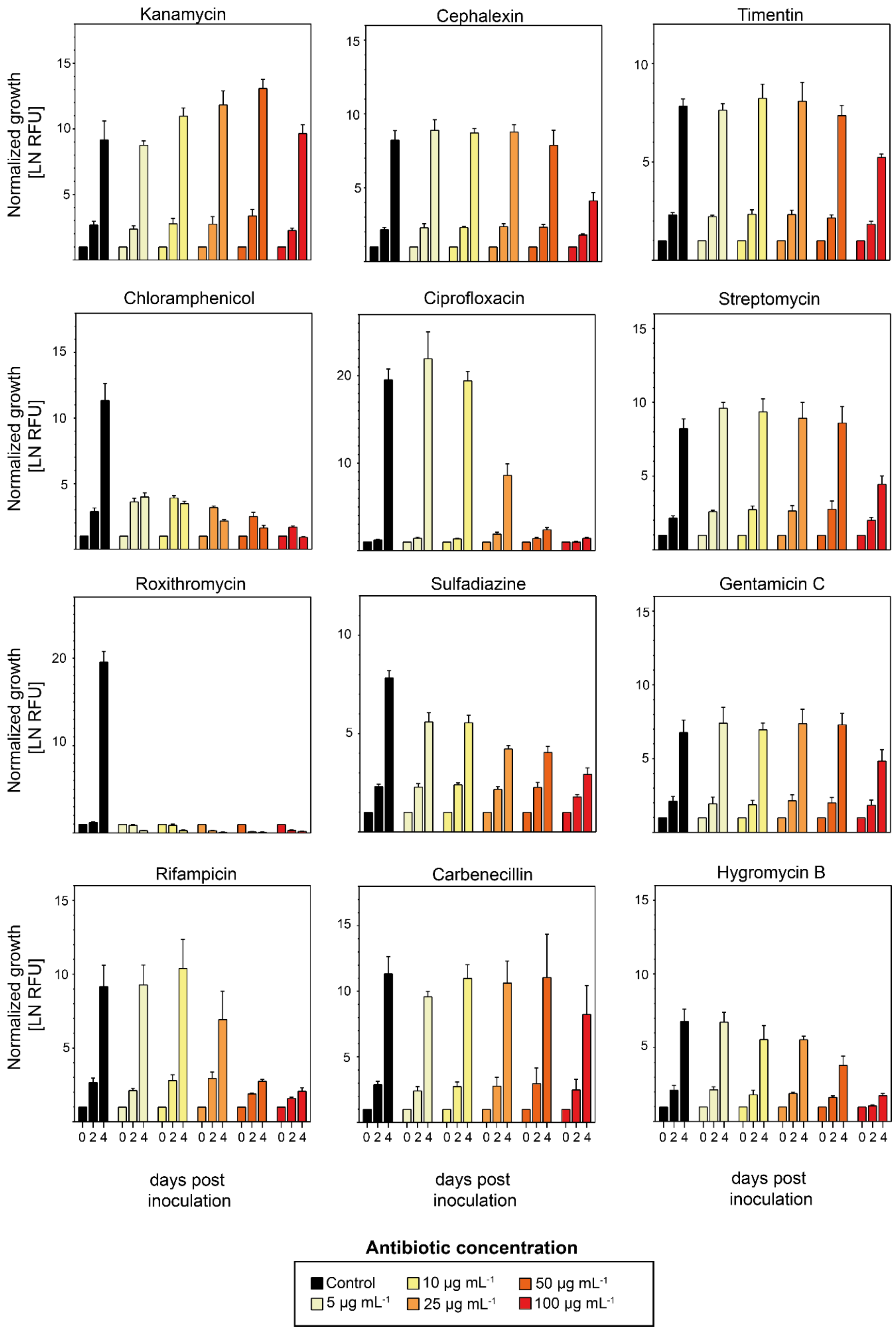

Figure S2. Antibiotic effects in the *S. marinoi* holobiont. Growth of *S. marinoi* cultures in the presence of various concentrations of antibiotics. The relevant antibiotic is indicated at the top of each graph, with the concentrations used as indicated below. Shown is the average growth rates of *S. marinoi* normalized at time zero and measured as RFU (n = 6, ± SD).

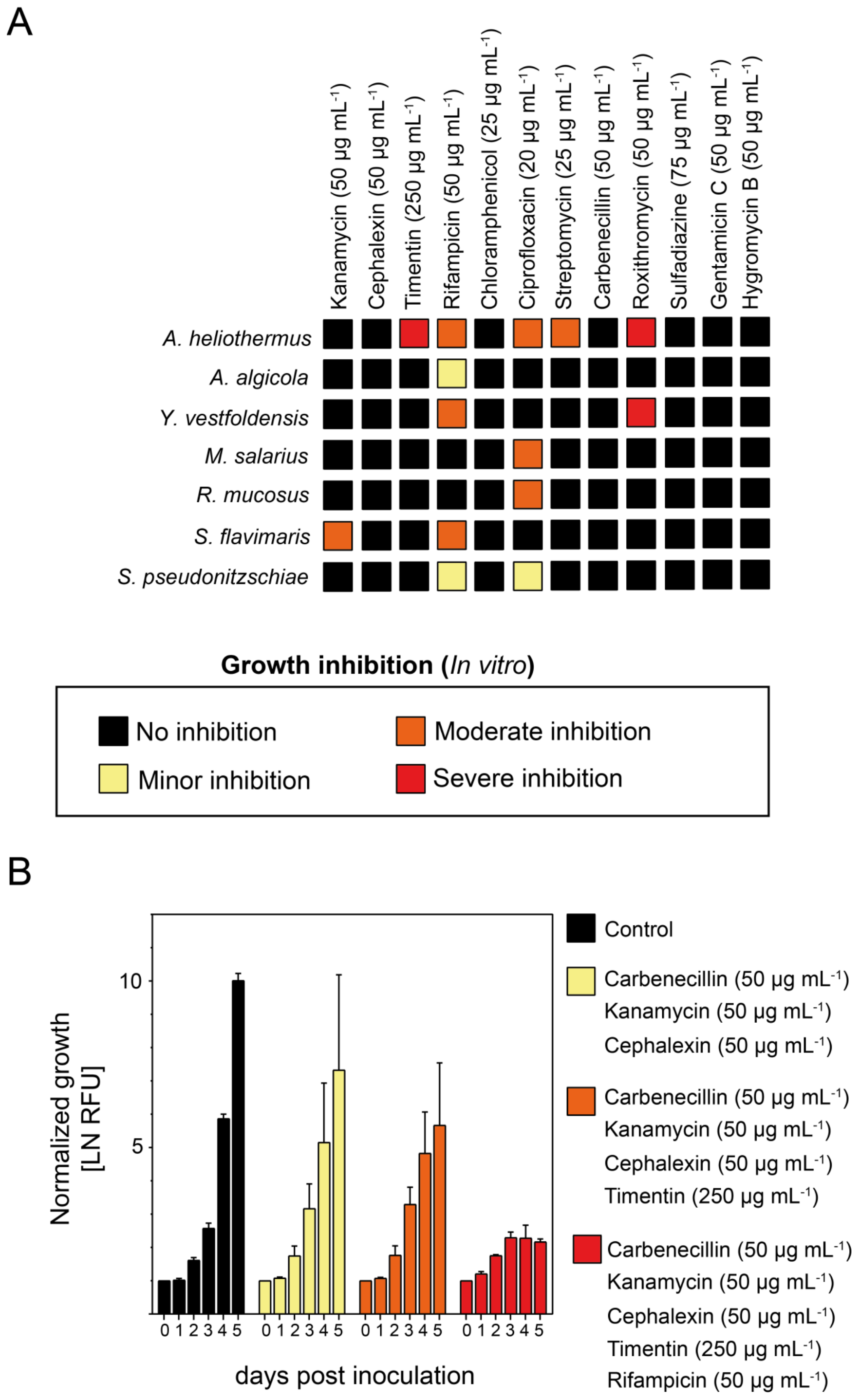

Figure S3. Antibiotic effectiveness on *S. marinoi* and its associated bacteria. (A) Relative inhibition by different antibiotics on the growth of each bacterial species isolated from *S. marinoi* cultures. The type and concentration of each antibiotic is shown above each column, with the bacterial species indicated on the left. The degree of growth inhibition (n = 6, ± SD) is shown by the color shading as indicated below. **(B)** The effect of three different antibiotic cocktails on the growth of *S. marinoi*. Growth was measured daily for five d, and plotted as average growth normalized at time zero and measured as RFU (n = 6, ± SD). The antibiotic composition of each cocktail is shown on the right.

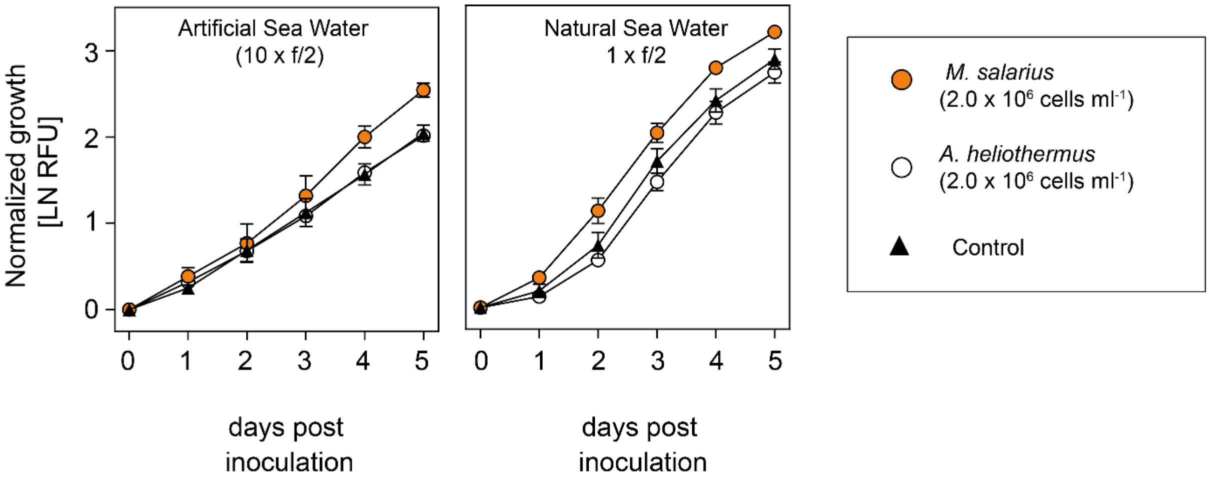

Figure S4. Growth promotion in different growth media. Growth of *S. marinoi* control (black triangles) and co-cultures (orange circles= *M. salarius*, white circles = *A. heliothermus*) in either Artificial sea water with 10 x f/2 addition or standard f/2 based on natural sea water. Shown are time zero normalized average LN RFU ($n = 4 \pm$ SD).

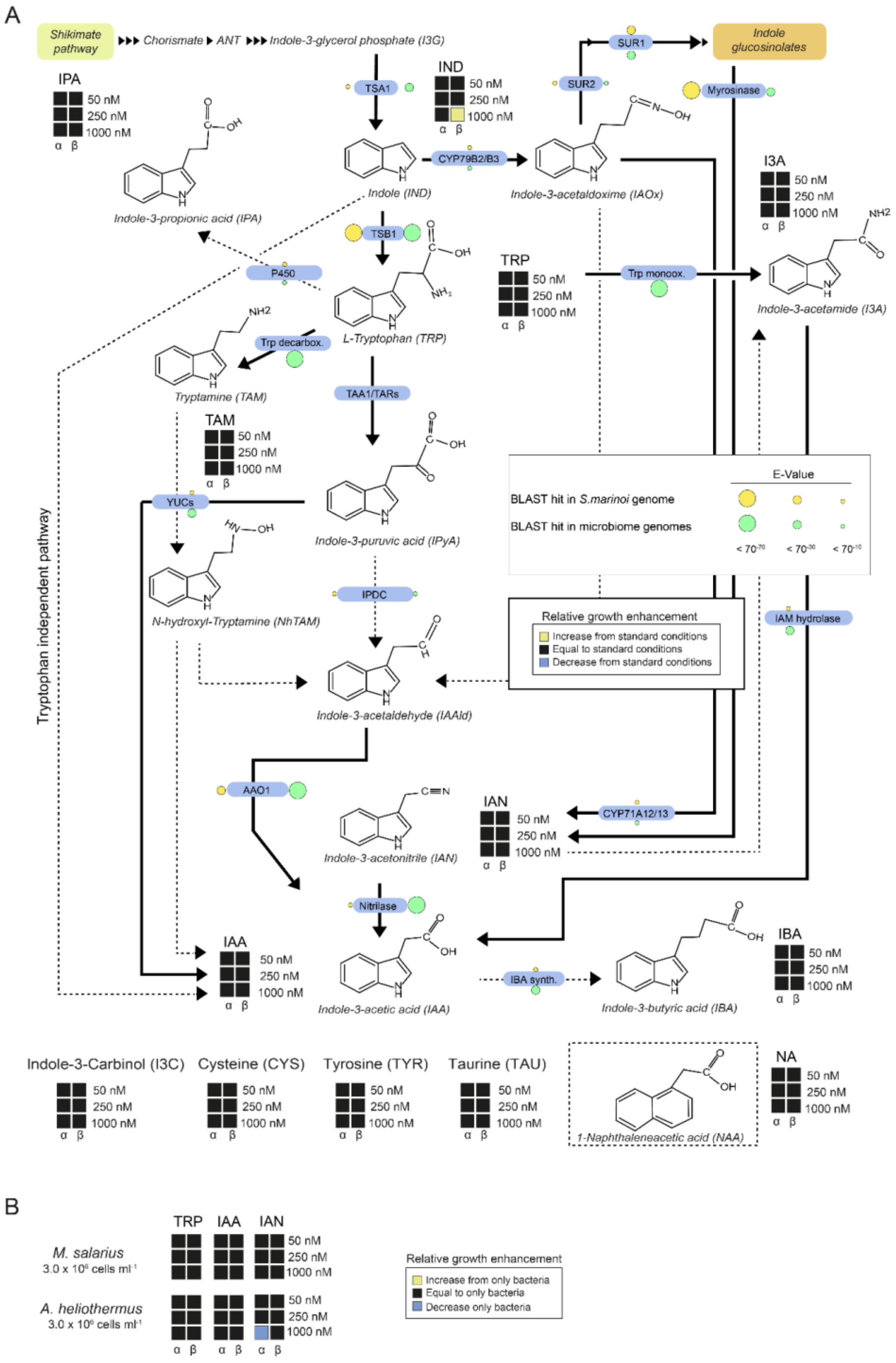

Figure S5. Auxin biosynthetic pathway and growth promotion. Shown in (**A**) are the known biosynthetic pathways of various auxins and the presence of genes encoding proteins performing each respective enzymatic step in either *S. marinoi* (yellow) or the microbiome (green). Each indicated compound was added to *S. marinoi* cultures in the concentrations shown and growth was monitored. The growth rate was compared by multiple t-tests in α (0-24h) and β (24-48h). Positive change from non-addition is shown as a color change (yellow) of the square, no single compound in any concentration reduced the growth of *S. marinoi*. In (**B**), the growth promotion of *S. marinoi* with the addition of either *M. salarius* or *A. heliothermus* in combination with three selected compounds at three different concentrations. Shown are the significant changes from that of only adding bacteria. Decrease in growth promotion is shown as a blue square compared to no change (black square). No combination improved beyond adding just bacteria.

A

Genome characteristics

Bacterial species	Genome Size (Bp)	Plasmids	Prophages
Antarctobacter heliothermus (A.h)	5,331,190	3	1+1 (plasmid) intact regions
Arenibacter algicola (A.a)	5,857,781	1	None
Yoonia vestfoldensis (Y.v)	3,987,360	2	2 intact regions
Marinobacter salarius (M.s)	4,630,160	1	1 partial region
Roseovarius mucosus (R.m)	4,381,426	2	3 intact regions
Sphingorhabdus flavimaris (S.f)	3,479,724	0	1 intact regions
Sulfitobacter pseudonitzschiae (S.p)	5,121,602	7	1 intact region (plasmid)

B

Biotic interaction related genes

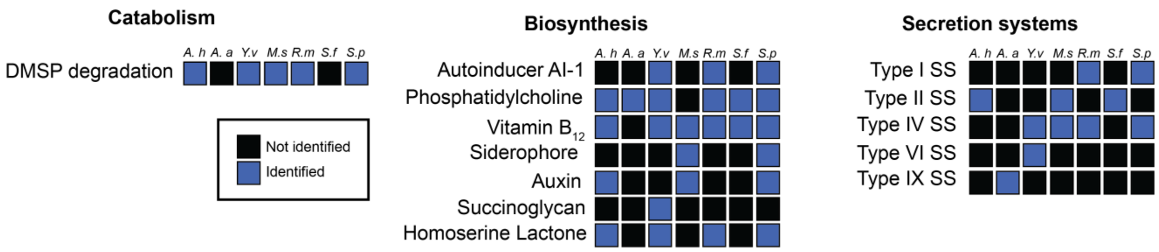

Figure S6. The bacterial genomes. Shown are the genome characteristics (**A**) of each of the seven bacterial genomes and the specific presence of genes involved in biotic-related interactions (**B**).

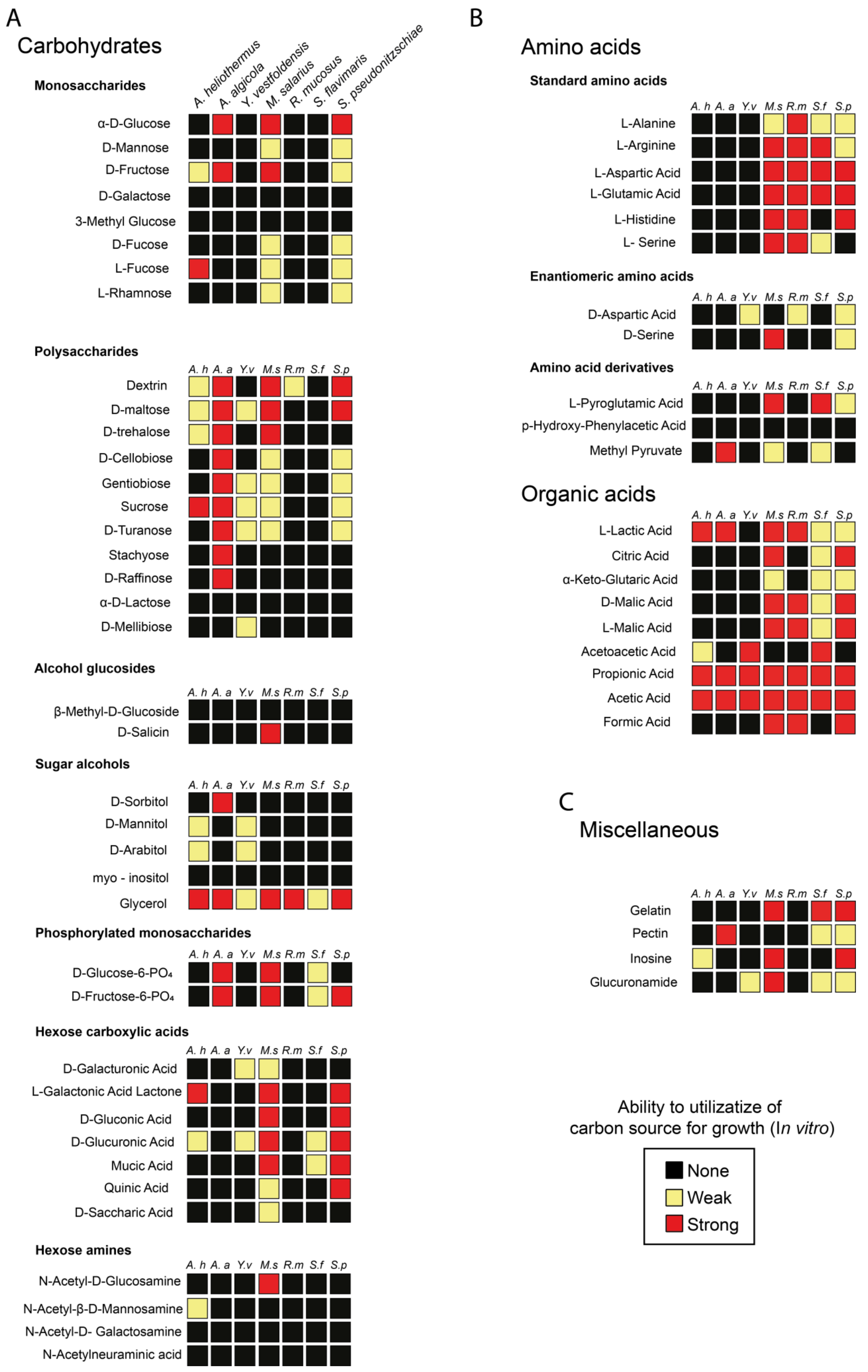

Figure S7. Growth preference of bacteria within the phycosphere. Shown is the capacity of each individual bacterial species to grow using the respective sources of carbohydrates (**A**), amino acids (**B**) and miscellaneous compounds (**C**) as indicated. Growth was scored as either no capacity (black square), weak capacity (yellow square) or strong capacity (red square).

Supplementary Table 1. Chemicals used in *S. marinoi* and bacterial growth assays. Working solutions (100 µM) were prepared in water from stock solutions for chemicals in the upper part in the table. Aliquots of these were added to the experimental cultures to the final concentrations indicated in the experiments. Stocks were kept in -20° until use.

Chemical	Short	CAS	Vendor	Solvent	Stock conc.
L-Cysteine	CYS	52-90-4	Merck	Water	0.1 M
Dimethylsulfoniopropionate	DMSP	4337-33-1	Merck	Water	0.1 M
Indole-3-acetamide	I3A	879-37-8	Merck	Methanol	0.05 M
Indole-3-Carbinol	I3C	700-06-1	Merck	DMSO	0.01 M
Indole-3-acetic acid	IAA	87-51-4	Merck	Ethanol	0.1 M
3-Indoleacetonitrile	IAN	771-51-7	Merck	Ethanol	0.1 M
Indole-3-butyric Acid	IBA	133-32-4	Merck	Ethanol	0.1 M
Indole	IND	120-72-9	Merck	Water	0.1 M
Indole-3-propoionic acid	IPA	830-96-6	Merck	Ethanol	0.1 M
1-Naphthaleneacetic acid	NA	86-87-3	Merck	Ethanol	0.1 M
Tryptamine	TAM	61-54-1	Merck	Ethanol	0.1 M
Taurine	TAU	107-35-7	Merck	Water	0.1 M
L-Tryptophan	TRP	73-22-3	Merck	0.1N HCl	0.1 M
L-Tyrosine	TYR	60-18-4	Merck	0.1N HCl	0.1 M
Kanamycin	KAN	25389-94-0	Duchefa Biochemie	Water	50 mg/mL
Cephalexin	CEF	15686-71-2	Duchefa Biochemie	Water	50 mg/mL
Timentin	TIM	61177-45-5	Biovision	Water	250 mg/mL
Rifampicin	RIF	13292-46-1	Duchefa Biochemie	Methanol	50 mg/mL
Chloramphenicol	CHL	56-75-7	Duchefa Biochemie	Ethanol	25 mg/mL
Ciprofloxacin	CIF	85721-33-1	Duchefa Biochemie	0.1N HCl	20 mg/mL
Streptomycin	STR	57-92-1	Duchefa Biochemie	Water	50 mg/mL
Carbenicillin	CARB	4697-36-3	Duchefa Biochemie	Water	50 mg/mL
Roxithromycin	ROX	80214-83-1	Merck	DMSO	15 mg/mL
Sulfadiazine	SULF	68-35-9	Merck	1M NaOH	75 mg/mL
Gentamicin C	GENT	1403-66-3	Duchefa Biochemie	Water	50 mg/mL
Hygromycin B	HYG	31282-04-9	Duchefa Biochemie	Water	50 mg/mL
